# Oligometastatic Breast Cancer: How to Manage It?

**DOI:** 10.3390/jpm11060532

**Published:** 2021-06-09

**Authors:** Vittoria Barberi, Antonella Pietragalla, Gianluca Franceschini, Fabio Marazzi, Ida Paris, Francesco Cognetti, Riccardo Masetti, Giovanni Scambia, Alessandra Fabi

**Affiliations:** 1 Medical Oncology 1, Regina Elena National Cancer Institute, IRCCS, 00144 Rome, Italy; vittoria.barberi@ifo.gov.it (V.B.); francesco.cognetti@ifo.gov.it (F.C.); 2 Scientific Directorate, Department of Woman and Child Health and Public Health, Fondazione Policlinico Universitario A. Gemelli, IRCCS, 00168 Rome, Italy; antonella.pietragalla@policlinicogemelli.it (A.P.); giovanni.scambia@policlinicogemelli.it (G.S.); 3 Comprehensive Cancer Center, Multidisciplinary Breast Unit, Fondazione Policlinico Universitario Agostino Gemelli IRCCS, Università Cattolica del Sacro Cuore, 00168 Rome, Italy; gianluca.franceschini@policlinicogemelli.it (G.F.); riccardo.masetti@policlinicogemelli.it (R.M.); 4 UOC Radiotherapy, Department of Imaging Diagnostic, Fondazione Policlinico Universitario A. Gemelli, IRCCS, 00168 Rome, Italy; fabio.marazzi@policlinicogemelli.it; 5 Department of Woman and Child Health and Public Health, Fondazione Policlinico Universitario A. Gemelli IRCCS, 00168 Rome, Italy; ida.paris@policlinicogemelli.it; 6 Unit of Precision Medicine in Breast Cancer, Scientific Directorate, Department of Woman and Child Health and Public Health, Fondazione Policlinico Universitario A. Gemelli, IRCCS, 00168 Rome, Italy

**Keywords:** oligometastatic breast cancer, locoregional therapy, CDK4/6 inhibitors, multidisciplinary

## Abstract

Breast cancer (BC) is the most frequent cancer among women and represents the second leading cause of cancer-specific death. A subset of patients with metastatic breast cancer (MBC) presents limited disease, termed ‘oligometastatic’ breast cancer (OMBC). The oligometastatic disease can be managed with different treatment strategies to achieve long-term remission and eventually cure. Several approaches are possible to cure the oligometastatic disease: locoregional treatments of the primary tumor and of all the metastatic sites, such as surgery and radiotherapy; systemic treatment, including target-therapy or immunotherapy, according to the biological status of the primary tumor and/or of the metastases; or the combination of these approaches. Encouraging results involve local ablative options, but these trials are limited by being retrospective and affected by selection bias. Systemic therapy, e.g., the use of CDK4/6 inhibitors for hormone receptor-positive (HR+)/HER-2 negative BC, leads to an increase of progression-free survival (PFS) and overall survival (OS) in all the subgroups, with favorable toxicity. Regardless of the lack of substantial data, this subset of patients could be treated with curative intent; the appropriate candidates could be mostly young women, for whom a multidisciplinary aggressive approach appears suitable. We provide a global perspective on the current treatment paradigms of OMBC.

## 1. Introduction

Breast cancer is the most frequent cancer among women and represents the second leading cause of cancer-specific death [1]. Metastatic breast cancer (MBC) includes about 6% of cases of de novo disease, and about 20–30% of early-stage cancers recurred at distant sites [2]. The behavior of stage IV breast cancer may differ, depending on the biology of the tumor, the likelihood of spreading to certain sites (e.g., bone in hormone receptor-positive disease), and the disease burden. A subset of patients with MBC presents limited disease, termed ‘oligometastatic’ breast cancer (OMBC) [3]. 

The concept of oligometastases represents a condition midway between locoregionally confined cancer and disseminated disease, in which tumor burden is low and the number of affected organs is limited, typically with 1 to 5 secundarisms [4,5,6,7,8]. 

Even though the incidence of OMBC is not clearly defined (1–10%), it seems that a considerable amount of all new MBC presents as oligometastatic. A tri-institutional retrospective analysis of 2249 patients with stage I–III disease who had first treatment failure showed that 21.9% were characterized by oligometastatic disease [9]. This boundary between oligo- and polymetastatic disease is increasingly recognized because of treatment and survival implications [3]. 

Given the likelihood of limited spread, it is possible to achieve longer survival, and, in 2–3% of cases, cure, with aggressive metastasis-directed therapy [5,8]. 

Moreover, OMBC is characterized by its chronicity and evolvement: primary cancer may present synchronous limited metastases, or the primitive tumor over time can develop a few metachronous metastases. We define oligorecurrence as the development of metachronous oligometastases with a controlled primary site [10], whereas oligoprogression represents a condition where a limited number of metastases progress, while all other sites of the disease remain stable, commonly during systemic treatment [11,12]. This distinction is representative of different scenarios and related prognosis, and it has a clinical implication in terms of survival [4].

For example, the patients with oligometastatic disease included in the previously cited study present a significantly longer overall survival (OS) as compared to polymetastatic patients with a follow-up of more than three years.

Prior reviews on oligometastatic disease investigated the effect of local techniques, namely surgical and radiotherapy. Recently, new techniques directed to disease biology provide information about next-generation treatment strategies, leading to a deeper biological understanding of OMBC and related treatment options. We provide a global perspective on the current treatment paradigms of OMBC [3].

## 2. Options for Treatment of Oligometastatic Breast Cancer

**Figure 1:**
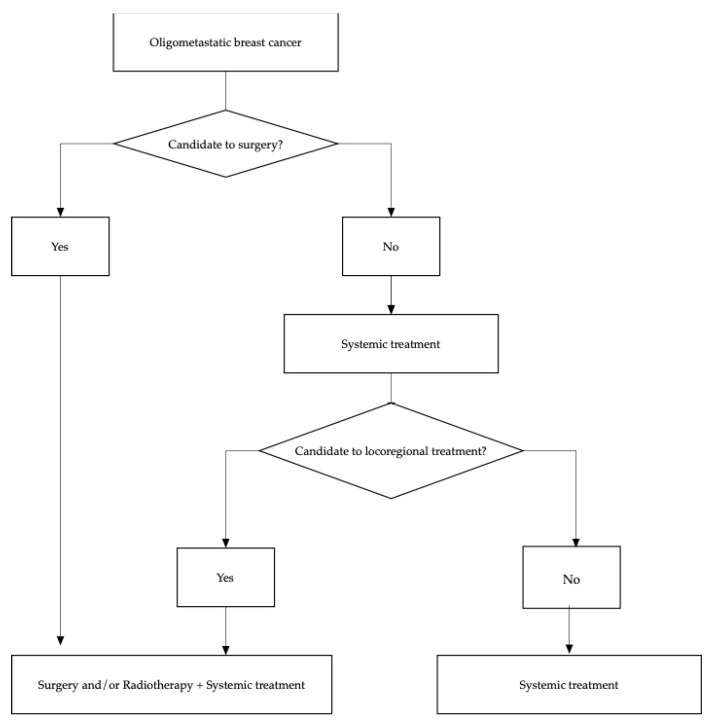
Diagram flow of therapeutic options in oligometastatic breast cancer.

The oligometastatic disease can be managed with different treatment strategies to achieve long-term remission and eventually cure. In Figure 1 a flow chart of treatment options is presented.

Several approaches are possible to cure the metastatic disease: locoregional treatment of the primary tumor and the metastases; systemic treatment, including target-therapy or immunotherapy, according to the biological status of the primary tumor and/or of the metastases; or the combination of these approaches [13].

Locoregional options both of the primitive tumor and of the metastases lead to long-lasting remissions reported in several case series; however, unlike other tumor entities, prospective data are lacking [13].

### 2.1. Surgery

In oligometastatic cancer, several trials involve surgery [14] (Table 1). The role of surgery in metastatic disease is unknown in terms of prognosis. Retrospective analyses demonstrate that patients who underwent surgery on the primitive tumor show a better prognosis compared to those who received only systemic therapy [15,16]. 

To corroborate a possible role of local treatments for the prognosis at the beginning of the metastatic disease, there is evidence that a multidisciplinary approach (surgery + radiotherapy, axillary dissection) is better for locoregional control of the disease, despite it being only surgery of the mammary node/mastectomy [17]. However, the findings of these studies are weakened due to selection bias: for example, patients with a less extended metastatic disease and/or who are responsive to medical treatments have more opportunities to undergo surgery on the primitive tumor than those who present a more advanced disease and/or who are less responsive to medical treatments. 

In the literature, three randomized trials evaluated the efficacy of surgery in MBC at the beginning of the disease.

In Tata Memorial Trial [18], among 350 women who enrolled, 173 underwent surgery and medical treatment and 177 received only medical treatment. This trial demonstrates that there are no differences in OS between the two groups. Surgical treatment is related to a better locoregional PFS, but also a worse DPFS (distant progression-free survival).

In the MF0701 [19] study, of 274 women who were enrolled, 138 underwent surgery and systemic treatment, while 136 were administered only systemic treatment. Patients with HR+ could receive hormone therapy. The protocol permitted upfront randomization (before the beginning of medical treatment) and the option of surgery on the primitive tumor during the local progression in the systemic treatment group. This trial showed a significant increase in median survival in those patients who underwent surgery upfront (46 vs. 37 months, HR 0.66 *p* < 0.005). An analysis of the subgroups showed that the survival was superior to locoregional treatment in women with luminal tumors, age < 55 years, and solitary bone metastases.

ECOG-ACRIN E 2108 [20] studied 258 patients with de novo MBC with no progression after 4–8 months of systemic treatment that were randomized to continue systemic treatment or to receive radical locoregional treatment (surgery with free margins and subsequent radiotherapy, if indicated). About 60% presented an HR+/HER-2 negative tumor, 26% HER-2+, 15% were triple negative. In addition, 37% of these patients presented only bone metastases. The survival analysis showed no difference in OS and PSF in the two cohorts in the general population. The subgroup analysis suggests a possible detrimental effect of locoregional treatment in the subgroup of patients with triple-negative BC. Thus, even though we observed an increase of 2.5 times in the risk of locoregional progression in patients who received systemic therapy without locoregional treatment, there is no benefit in terms of quality of life from locoregional treatment.

Moreover, a prospective cohort trial [21] shows that in patients who have responded to first-line treatment, surgery on the primitive tumor does not improve PFS and OS, so that the predominant prognostic role is given by medical treatments, histopathologic features, and tumor burden. Conclusively, in patients with de novo MBC, the surgery approach has a palliative role (e.g., ulcerative lesions). In the absence of results of the effectiveness in OS, this procedure is considered in selected cases and after discussion with the patient.

There are three other randomized trials, one of which has finished the accrual, and it could furnish other elements to the argument. 

In clinical practice, surgery is reserved for vertebral metastases with medullary compression, pathological fractures, pleural or pericardial effusion, and single visceral metastasis (e.g., liver, lung, brain).

In this regard, the resection of liver metastases in MBC is little explored, although in other tumors such as colorectal cancer it is widely recognized [6].

Different case series [6,22,23,24,25,26,27,28,29,30,31,32,33,34,35,36] show different survival rates (22–61 months) for liver metastases resection. A monocentric experience with 51 patients reported a 16% increase of 10-year OS rate [26]; 8.9% of these patients never presented any recurrence after surgery. However, this result is affected by a selection bias of the sample: the resection, but also the indolent course of the disease, the specific genetic profile of the tumor, and the ability of subclones to metastasize to a certain organ likely play a crucial prognostic role. Therefore, these reports need confirmation with prospective randomized trials [37]. 

A prospective data collection of 41 patients, who underwent liver metastases resection, revealed that positive resection margins and a short disease-free interval until the detection of liver metastases may lead to poor long-term survival [38]. Comparable results can be assumed for pulmonary lesions metastasectomy [39,40]: a short disease-free interval, the presence of several metastases, incomplete resection of them, and a non-luminal subtype are considered negative prognostic factors [41]. 

In summary: in OMBC, surgery on the metastases is still experimental because there are no data from prospective randomized trials with large samples. In addition, OMBC, even the indolent behavior, is a widespread disease, where local treatments alone could not be sufficient. However, these preliminary results may identify subgroups of patients with more favorable outcomes and for whom the surgery could lead to long-term survival [13].

### 2.2. Radiotherapy

Patients with oligometastatic disease or with oligorecurrence in a single area could be treated with local therapy such as stereotactic body radiotherapy (SBRT), even associated with chemotherapy. Possible target lesions include brain, lung, liver, and lymph nodes.

Oligorecurrent metastases in the brain, lung, and liver can be definitively treated with SBRT. Instead, there are some controversies regarding lymph node oligometastases, thus further phase III trials are needed [42].

The use of stereotactic ablative radiotherapy (SABR) produces favorable outcomes, since it presents high accuracy to the target lesion, very conformal dose distributions, and delivers a highly ablative dose over a treatment duration of 1–5 treatments maximum. 

Several works strengthen the use of SABR in OM disease, mostly randomized controlled trials (RCTs) [4].

Patients with a limited number of brain metastases and controlled extracranial disease may benefit from locoregional treatment combined with systemic therapy, which crosses the blood–brain barrier. Currently, stereotactic radiosurgery (SRS) is the recommended option for resected cavity and non-resected brain metastases [43] and achieves longer overall survival (OS) compared to whole-brain palliative irradiation [44,45].

Concerning lung OM disease, stereotactic techniques demonstrate a 2-year local control rate of 77.9% and a 2-year OS of 53.7%, according to a systematic review [46].

At the same time, a regional nodal recurrence after conservative breast treatment affects about 1% to 5.4% of patients with early-stage breast cancer [47,48,49]. A phase II study with SBRT or intensity-modulated radiation therapy for OMBC showed encouraging results [50]. Even though the principal site of metastases was the bone, several cases of lymph node metastases were treated with SBRT or intensity-modulated radiation therapy, without reporting severe toxicity. Furthermore, 90% of patients with oligorecurrence had an objective response to salvaging radiotherapy and the 3-year treated tumor control rate was 93% [51]. However, despite the lack of reports about SBRT for oligorecurrent lymph node metastases of breast cancer, this subgroup of patients seems to be well suited for SBRT, especially those who did not receive previous irradiation, because of the indolent behavior of the disease. Nonetheless, patients should be carefully monitored over time, because of the risk of late toxicities.

The research is moving forward, with an ongoing randomized phase II/III trial (NRG-BR002), which evaluates the role of these techniques in OMBC [42,52].

### 2.3. Systemic Treatments

Systemic treatment remains a milestone in the management of metastatic breast cancer.

Considering hormone receptor (HR) positive, HER2-negative metastatic breast cancers, certainly CDK4/6 inhibitors in combination with endocrine therapy have changed the paradigm of the treatment [53].

Concerns about the difference among the three CDK4/6 inhibitors involve the significant OS improvement, demonstrated from MONALEESA-3, MONALEESA-7, and MONARCH-2 trials, but not reported in PALOMA-1, PALOMA-3, and MONALEESA-2 trials [54,55,56,57,58,59].

As a result, a meta-analysis of all these randomized controlled trials evaluated the OS improvement among Palbociclib, Ribociclib and Abemaciclib, and focused on the efficacy of these compounds in some relevant subgroups of patients. 

Of 5862 patients from MONALEESA-2, MONALEESA-3, and MONALEESA-7 trials, 2429 presented visceral (lung or liver) disease, 929 had bone-only disease, and 2504 had visceral and bone disease. Of 2845 patients, grouped by the number of metastases, 782 had only one metastatic site, 635 two, and 1428 three or more. The pooled results of the meta-analysis showed no heterogeneity for all these subgroups, with a statistically significant improvement in PFS with a similar hazard ratio [60]. 

Therefore, this meta-analysis demonstrates that CDK4/6 inhibitors plus endocrine therapy are beneficial in terms of PFS, regardless of the presence of visceral metastases, the number of metastatic sites, and the length of the treatment-free interval. Consequently, the pooled estimate for the overall population is also feasible for OMBC patients [60].

However, in luminal breast cancer, even after a first-line systemic treatment, OM disease could be persistent; therefore, due to the introduction and approval from FDA and EMA of Alpelisib, it is advisable to test the presence of PIK3CA mutation. Patients with PIK3CA mutation may benefit from Alpelisib plus Fulvestrant association, both with bone metastases and visceral metastases, as shown by the subgroup analysis of the SOLAR-1 study [61]. Instead, patients without the expression of PIK3CA mutation should receive a further line of hormonal treatment; this can be Everolimus plus Exemestane or Fulvestrant alone or, in selected patients, chemotherapy; confirmed data about the use of CDK4-6 inhibitors beyond progression are still unknown, and to date there are ongoing phase III studies comparing Alpelisib plus Fulvestrant versus Fulvestrant alone (CBYL719C2303 study-EPIK-B5). 

In summary, a key role in the OMBC treatment is maintaining hormonal target therapy, reserving chemotherapy in cases of visceral crisis or widespread disease. 

Unlike the luminal BC, often HER-2-like and triple-negative tumors have a different presentation since they have more aggressive behavior. Therefore, in these subtypes the strategy overlaps with a polymetastatic disease: in case of an HER-2 like OMBC, the use of anti-HER-2 molecules remains the first goal; instead, the current targets for triple-negative tumors are PD-L1 and BRCA mutations, and the use of Atezolizumab plus Nab-paclitaxel and Olaparib, respectively, showed better outcomes in terms of PFS and quality of life [62,63]. 

### 2.4. Combination of Radiotherapy and Systemic Treatment

Although CDK4/6 inhibitors are largely involved in the treatment of MBC, preliminary findings suggest a possible synergic effect of these compounds when combined with radiotherapy, especially in OM disease [64]. 

CDK4/6 inhibitors can act as a DNA double-strand break repair inhibitor, thus amplifying the anticancer effect of RT [65].

Therefore, the simultaneous administration of a radio-sensitizing drug could significantly improve symptoms and disease control. Despite the potential benefit of this combination, there is little literature on this topic, and clinicians could be frightened, since the radio-sensitizing effect may also increase the toxicity, involving healthy tissues as well [66,67]. The consequence might lead to, on one hand, improperly interrupting the systemic treatment or the radiotherapy.

Table 2 shows the preliminary results from small patient samples with the combination of CDK4/6 inhibitors with RT. 

Hans et al. described five patients treated with Palbociclib and concurrent palliative RT without severe toxicity [68]: all patients experienced pain relief, but follow-up time and local control were not reported. 

Meattini et al. described five patients treated with Ribociclib and concurrent palliative RT for bone metastases [69]: two patients developed grade 3–4 toxicity (one neutropenia and one vomit and diarrhea) and two needed temporary suspension of Ribociclib; radiotherapy was never suspended. At a 3-month assessment, three stable diseases and two partial responses were observed. 

Chowdary et al. evaluated 16 patients treated with Palbociclib and RT for symptomatic metastases [64]. No side effect differences were found compared to the use of Palbociclib alone; all patients experienced prolonged pain control, and no local failures were described. However, only 31.3% of patients did not interrupt Palbociclib during the RT, while the other patients suspended the CDK4/6 inhibitor 14 days before or after RT, with a median interval of 5 days. 

Ippolito et al. analyzed 16 patients treated with Palbociclib or Ribociclib concomitant to RT [70]. First, 68.7% of patients received palliative RT for bone metastases with a median dose of 30 Gy, while the remaining with OM disease were treated with higher doses (median 50 Gy). At 6.3 months follow-up, the only toxicity reported was neutropenia, apparently not worsened by radiotherapy, because it had already existed during the previous cycles of systemic treatment. Patients with bone metastases experienced all pain relief; the other subgroup developed complete responses (two patients with visceral and/or soft tissue), partial responses (two patients with bone disease), and stable disease (one patient with bone involvement) [71,72,73,74,75,76].

Two other retrospective analyses evaluated risks and benefits from the concomitant therapy with CDK4/6 inhibitors and RT. In one experience 16 patients under treatment with Palbociclib and radiotherapy were studied. At a follow-up of 14.7 months, none reported relevant acute or late toxicities: all reported that side effects were mild. All the patients achieved pain relief, and no local failures were developed [64]. The second study analyzed 18 patients treated with radiotherapy and concomitant CDK4/6 inhibitors for bone involvement. The hematologic toxicity was mild during the end of RT and the subsequent cycles of systemic treatment (grade 3–4 neutropenia) [72]; the other relevant side effect was grade 1 gastrointestinal toxicity. Three months after the end of RT, 88.9% of patients experienced pain relief, with no pain recurrence. With a median follow-up of 13.7 months, only one patient developed local recurrence. This study involves the largest cohort with concomitant CDK 4/6 inhibitors and RT published, but numbers are still limited. 

These preliminary works suggest that the combination of CDK4/6 inhibitors and RT, particularly on bone metastases, is safe, with limited toxicities in terms of time and grade. The hematologic toxicity is comparable between the combination of these approaches and the medical treatment alone, while the gastrointestinal side effects could be more relevant; therefore, clinicians should be careful in case of RT of the abdominal or pelvic area. 

Although the results of these trials are limited by the small number of the sample, the clinical and radiological outcomes are promising. Future studies with a larger population and a longer follow-up will validate these results [64,77].

## 3. Conclusions

Even though metastatic breast cancer is considered incurable, OMBC presents a better prognosis [78].

Regardless of the lack of substantial data, this subset of patients could be treated with curative intent, mostly young women for whom a multidisciplinary aggressive approach appears suitable [3,78]. 

For these patients with a favorable nature for their disease, a multidisciplinary aggressive approach might improve survival [78].

Specifically, a combination of local and systemic treatment can achieve such long-term effects [13].

Local ablative options (radiotherapy/surgery) play a key role in this setting, as can be assumed from retrospective trials, but these encouraging results need confirmation by prospective randomized studies [78]. 

Moreover, preliminary data suggest an increase of disease-free survival after surgery on distant metastases; however, the selection of the appropriate candidates concerns the biology of the disease, and unfortunately, valuable comparative data are still missing. For this reason, surgery on breast cancer metastases remains an experimental approach.

Systemic therapy, e.g., the use of CDK4/6 inhibitors for HR+/HER2 negative BC, leads to an increase of PFS and OS in all the subgroups, with favorable toxicity.

Therefore, combined strategies increase the probability of producing results such as tumor-size reduction, long-lasting responses, and, eventually, cure [79].

All of these treatment strategies present a higher rate of success when the metastatic disease is detected early, so it is crucial to involve modern imaging equipment and liquid biopsies to model a personalized and multidisciplinary treatment [13].

## 4. Future Directions

The lack of strong data concerning the management of OMBC clearly emerges, due to the quality and heterogeneity of the systematic reviews and meta-analyses.

However, the increasing interest in the OM phenotype is emerging, and several prospective phase II/III randomized controlled trials involving new strategies for OMBC are ongoing (Table 3). A phase III study in the Netherlands (NCT01646034) is evaluating the role of high-dose chemotherapy with carboplatin, thiotepa, and cyclophosphamide in homologous recombination-deficient OMBC, since it seems that these tumors are particularly sensitive to alkylating agents which disrupt double-stranded DNA. Several trials are assessing the use of SABR and/or traditional surgery associated with systemic therapy in the first-line setting for newly diagnosed OMBC (e.g., CLEAR, NCT03750396; STEREO-SEIN, NCT02089100; NCT02364557). For instance, a pilot phase I study in Australia is evaluating the role of SABR followed by 6 months of anti-PD1 therapy with pembrolizumab, intending to show both safety and enhanced immune activation (BOSTON-II, NCT02303366).

The comparison of these trial results is weakened by the different definition of ‘oligometastatic disease’, which could include from two to five distant lesions. For further future studies, it would be reasonable to employ a universal definition of ‘oligometastatic’ within the breast cancer investigative community [3].

## Figures and Tables

**Table 1:** Randomized trials that evaluate the efficacy of surgery in MBC.

Trial	Number of Patients	Site of Metastases	Biological Subtype	Site of Surgery	Outcome
Tata Memorial, NCT00193778	350	Bone and/or visceral	HR+ /HER2− HR+/HER2+	- Modified radical mastectomy - Breast-conserving surgery - Palliative surgery upon progression	1. No differences in OS 2. Better locoregional PFS FOR surgery 3. Worse DPFS for surgery
MF0701, NCT00557986	274	Bone and/or lung and/or liver	HR+ 85.5% HER2+ 30.4% TN 7.3%	- Breast conserving surgery - Metastasectomy - Axillary lymph node dissection	1. Increase in median survival for surgery upfront 2. Superior survival for locoregional treatment in women with luminal tumors, age < 55 years, and solitary bone metastases
ECOG-ACRIN E 2108, NCT01242800	258	Bone and/or any organ system, including CNS	HR+/HER2− 60% HER2+ 26% TN 15%	- Breast-conserving therapy - Total mastectomy - Palliative surgery	1. No difference in OS and PFS 2. Possible detrimental effect of locoregional treatment in TN mBC 3. Increase of 2.5x risk of locoregional progression in patients who received systemic therapy without locoregional treatment
TBCRC 013, NCT00941759	127	Bone and/or any organ system, including CNS	HR+/HER2– HR+/HER2+ HR−/HER2+ HR−/HER2−	- Elective breast surgery - Palliative breast surgery	1. No improvement of PFS and OS for surgery in patients who have responded to first-line treatment

**Table 2:** Trials that evaluate the efficacy of concomitant RT and CDK4/6-i in MBC.

Trial	Number of Patients	CDK4/6-I	Outcome
Hans et al.	5	Palbociclib	5 pain relief 1 stable disease
Meattini et al.	5	Ribociclib	3 stable disease 2 partial response
Chowdary et al.	16	Palbociclib	16 pain relief 0 local failures
Ippolito et al.	16	Palbociclib Ribociclib	16 pain relief 2 complete responses 2 partial responses 1 stable disease
Mudit et al.	16	Palbociclib	16 pain relief 0 local failures
Guerini et al.	18	Palbociclib Ribociclib Abemaciclib	16 pain relief 0 pain recurrence 17 local control 1 local recurrence

**Table 3:** Ongoing trials in oligometastatic BC.

Trial	Objective	Site of Metastases
NCT01646034	Role of high-dose polychemotherapy in HRD OMBC	1 to 3 distant metastatic lesions, with or without primary tumor, local recurrence, or locoregional lymph node metastases, including the ipsilateral axillary, parasternal, and periclavicular regions
CLEAR, NCT03750396	Local treatment (including surgical resection, stereotactic body radiotherapy, palliative radiotherapy, and radiofrequency ablation) in addition to endocrine treatment as 1st line for HR+/HER2- OMBC	≤2 lesions in single organ or site (lung, bone, liver, adrenal glands, distant LNs)
STEREO-SEIN, NCT02089100	Role of metastases SBRT with curative intent in de novo oligometastatic disease	≤5 metastatic lesions (measurable or not) No brain metastases
NCT02364557	Use of SABR and/or traditional surgery in addition to standard of care systemic therapy in the first-line setting for newly diagnosed OMBC	≤4 metastases in lung, bone, spine, abdominal-pelvic (lymph node/adrenal gland), liver, mediastinal/cervical lymph node
BOSTON-II, NCT02303366	Role of SABR followed by 6 months of anti-PD1 therapy with pembrolizumab, intending to show both safety and enhanced immune activation	1 to 5 metastases No evidence of visceral metastases in liver or brain

## Data Availability

Not applicable.
